# Compound heterozygous GLI3 variants in siblings with thyroid hemiagenesis

**DOI:** 10.1007/s12020-020-02422-1

**Published:** 2020-07-21

**Authors:** Ewelina Szczepanek-Parulska, Bartłomiej Budny, Martyna Borowczyk, Katarzyna Zawadzka, Paweł Sztromwasser, Marek Ruchała

**Affiliations:** 1 Department of Endocrinology, Metabolism and Internal Diseases, Poznan University of Medical Sciences, 49 Przybyszewskiego Street, 60-355 Poznan, Poland; 2 MNM Diagnostics Sp. z o.o, 64 Macieja Rataja Street, 61-695 Poznan, Poland; 3 Department of Biostatistics and Translational Medicine, Medical University of Lodz, 15 Mazowiecka Street, 92-215 Lodz, Poland

**Keywords:** Thyroid hemiagenesis, Thyroid dysgenesis, *GLI3* gene, *GLIS3* gene, Whole-exome sequencing

## Abstract

**Purpose:**

Thyroid hemiagenesis (THA) is an inborn absence of one thyroid lobe of largely unknown etiopathogenesis, affecting 0.05–0.5% population. The aim of the study was an identification of genetic factors responsible for thyroid maldevelopment in two siblings with THA.

**Methods:**

We evaluated a three-generation THA family with two sisters presenting the disorder. Proband (Patient II:3) was diagnosed at the age of 45 due to neck asymmetry. Left lobe agenesis and nontoxic multinodular goiter were depicted. Proband’s sister (Patient II:6) was euthyroid, showed up at the age of 39 due to neck discomfort and left-sided THA was demonstrated. Affected individuals were subjected to whole-exome sequencing (WES) (Illumina, TruSeq Exome Kit) and all identified variants were evaluated for pathogenicity. Sanger sequencing was used to confirm WES data and check segregation among first-degree relatives.

**Results:**

In both siblings, a compound heterozygous mutations NM_000168.6: c.[2179G>A];[4039C>A] (NP_000159.3: p.[Gly727Arg];[Gln1347Lys]) were identified in the *GLI3* gene, affecting exon 14 and 15, respectively. According to the American College of Medical Genetics, variants are classified as of uncertain significance, and were found to be very rare (GnomAD MAF 0.007131 and 0.00003187). The segregation mapping and analysis of relatives indicated causativeness of compound heterozygosity.

**Conclusions:**

We demonstrated for the first time a unique association of THA phenotype and the presence of compound heterozygous mutations p.[Gly727Arg];[Gln1347Lys] of *GLI3* gene in two siblings.

## Introduction

Thyroid hemiagenesis (THA) is a congenital lack of one thyroid lobe, with an incidence of 0.05–0.5% in population [1]. Although THA was previously considered a benign thyroid maldevelopment, latest data suggest that it might be associated with an increased incidence of compensatory hypertrophy of a single thyroid lobe, nodular goiter, and possibly autoimmune thyroid diseases [2]. Familial occurrence of thyroid developmental anomalies (TDA), coincidence of TDA and several genetic syndromes together with an observation that THA is strain-dependent in rats suggest a genetic background [3]. The first genes suspected to be involved in thyroid embryogenesis were thyroid transcription factors [4]. However, mutations in these genes turned out to be causative only in a few patients with severe syndromic forms of TDA, mainly thyroid agenesis or ectopy, while etiopathogenesis of majority THA isolated forms remains unknown [5].

To date, in THA patients only a heterozygous mutation in *PAX8* gene was documented as genetic cause [6]. Recently, our group has demonstrated that proteasome genes mutations may be associated with THA phenotype [7]. Furthermore several uncertain changes, but no deleterious mutations in *HOXB3*, *HOXD3*, and *PITX2*, were detected. In another patient with THA, a microduplication of chromosome region 22q11.2 encompassing *TBX1* gene was found [1].

The aim of the study was to identify novel genetic factors potentially responsible for thyroid maldevelopment by extensive genetic analysis of two patients with a familial form of THA.

## Material and methods

### Patients

The studied family consisted of 13 members of which 2 sisters presented an isolated form of THA. Both patients showed agenesis of the left thyroid lobe. Although sisters were born before the introduction of TSH neonatal screening in Poland, they did not present any features of congenital hypothyroidism (CH) and were diagnosed due to suspicion of goiter at adult age.

**Fig. 1:**
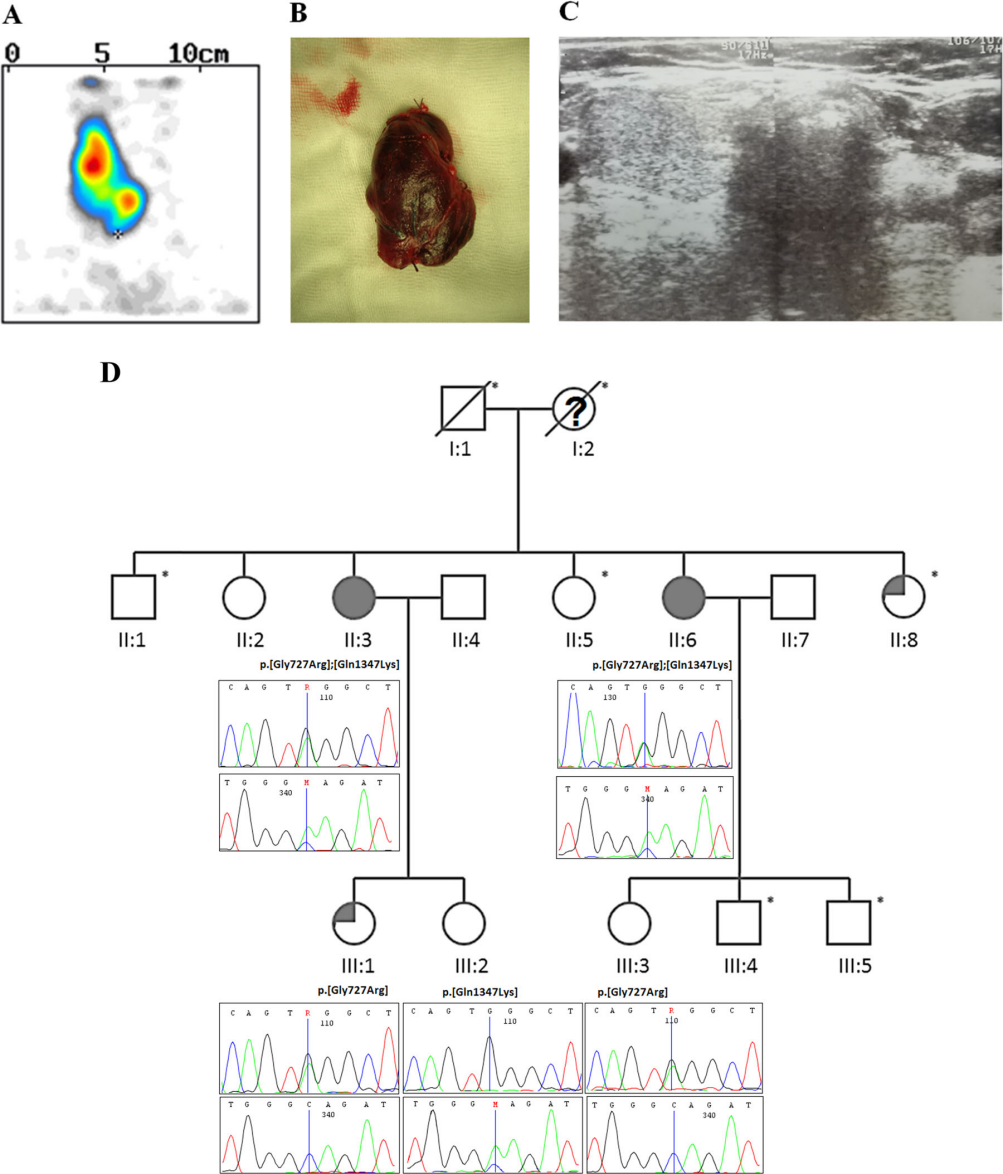
Tc99m scintiscan (**a**) and surgical specimen (**b**) obtained after complete thyroidectomy, depicting agenesis of the left thyroid lobe in the patient II:3. Agenesis of the left lobe in the patient II:6 demonstrated by thyroid ultrasound examination (**c**). **d** Pedigree of the THA family. Blackened symbols are presenting affected sisters, symbols with an asterisk indicating individuals who were not examined with US. The remaining patients were confirmed to have bilobed thyroid. Individual I:2—neck asymmetry was reported by family members therefore a question mark regarding THA status was used, II:8—thyroidectomy, multinodular goiter III:1—hypothyroidism, Hashimoto’s thyroiditis

Patient II:3 (proband) was referred to an endocrinologist at the age of 45 due to neck asymmetry. Thyroid ultrasound (US) and Tc99m scintiscan (Fig. 1a) revealed presence of the enlarged right lobe (volume 19.3 ml) with multinodular goiter and lack of the left lobe. She was euthyroid [TSH 0.755 uIU/ml (normal 0.27–4.2), free triiodothyronine FT3 4.54 pmol/l (normal 2.25–6.0), free thyroxine FT4 12.82 pmol/l (normal 9–20)]. Thyroid autoantibodies were within normal ranges. The result of fine-needle aspiration biopsy was consistent with benign colloid nodules. However, due to local symptoms and progression of the lesions in size during follow-up period, the patient was subjected to thyroidectomy (Fig. 1b). Histopathological examination revealed benign colloid goiter. She has two daughters, presenting normal thyroid on US.

Her sister (patient II:6) was diagnosed at the age of 39 due to neck discomfort. Thyroid US (Fig. 1c) and Tc99m scintiscan revealed presence of the enlarged right thyroid lobe of normal echogenicity (volume 14 ml). She presented clinical and biochemical euthyroidism with negative antithyroid autoantibodies. Patients’ offspring, a daughter and two sons, did not present any apparent thyroid disease. Unfortunately, sons refused to undergo genetic investigation.

Patients II:3 and II:6 have four other siblings (three sisters and one brother). One of those sisters had a sonographically normal thyroid; another sister (II:8) underwent thyroidectomy due to multinodular goiter in a normally developed bilobed gland. The third sister (II:5) and brother had no apparent thyroid disease but refused to undergo in-depth thyroid diagnostics. At the moment of diagnosis, both grandparents were dead (I:1, I:2), with a negative history of thyroid diseases (Fig. 1d).

### Genetic testing

**Fig. 2:**
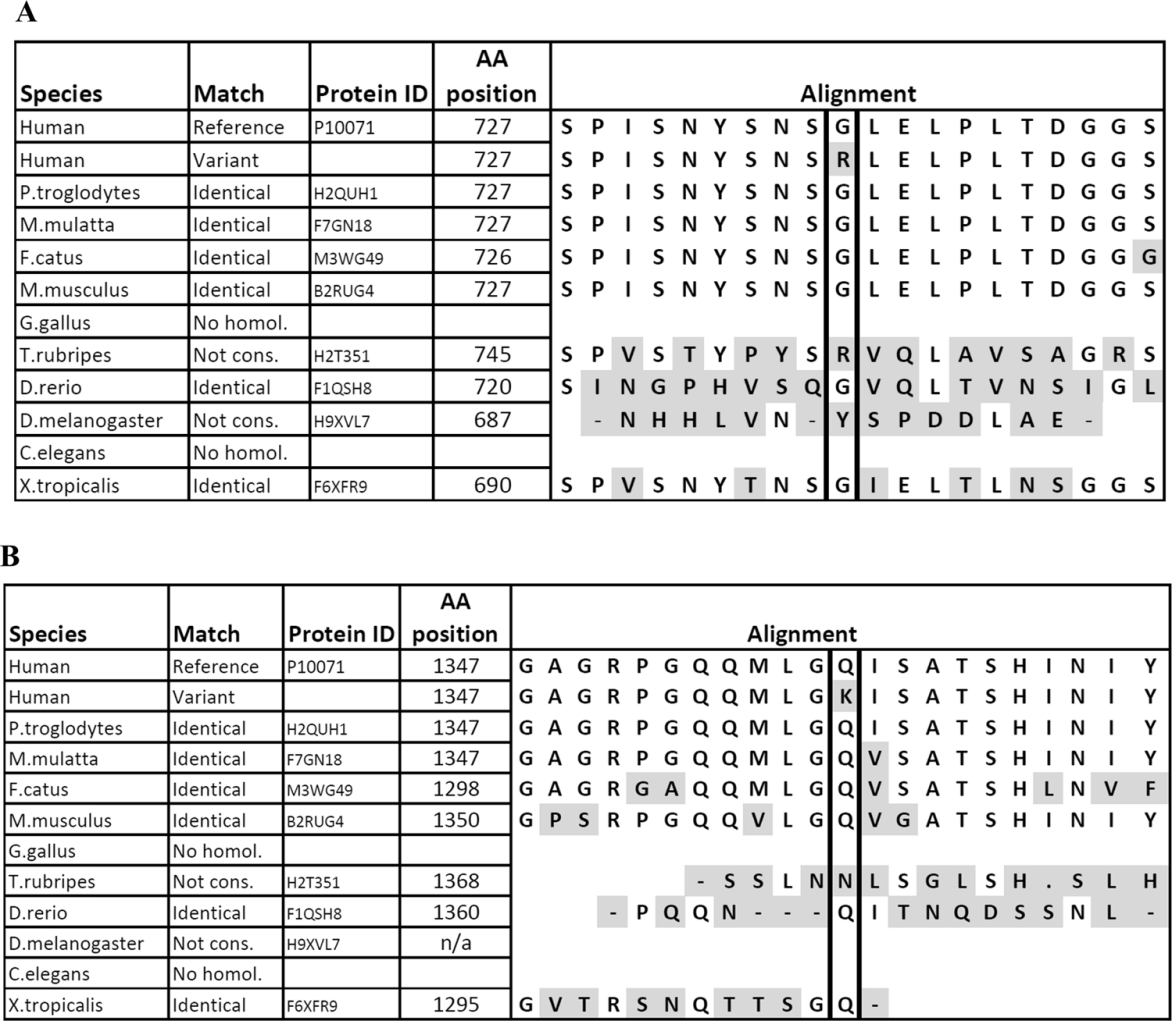
A demonstration of the phylogenetic conservation of Gly727 (**a**) and Gln1347 (**b**)

DNA was isolated from peripheral blood with the use of standard procedure. At first, the DNA of affected individuals (II:3, II:6) was screened. We performed Sanger sequencing of the coding and neighboring intronic regions of the genes which role in the thyroid embryogenesis was documented in previous studies. Conventional sequencing of the following genes was performed: *TTF1 (NKX2–1), TTF2 (FOXE1), PAX8, HHEX, SHH, TBX1, PSMA1, PSMA3, PSMD2, PSMD3*. As the initial search failed in detection of causative mutations, samples were subjected to whole-exome sequencing (WES) with the use of Illumina platform (TruSeq Exome Enrichment Kit, minimal mean depth 218×). Ninety-nine percent of the reads were aligned to hg19 using BWA v0.7.12, Picard v1.130, GATK v3.4.0, SnpEff v4.1g. For pathogenicity evaluation, the following algorithms and databases were used: Sift, PolyPhen2, dbNSFP, FATHMM, MutationTaster v2, PhenIX, HPO database, and population data from GnomAD v2.1.1. For the evolutionary conservation measurement we used PhyloP and phastCons (Fig. 2).

Patients have signed informed consent form for genetic studies and publication of this report. Bioethical Committee of Poznan University of Medical Sciences approved the study (approval number 524/18).

## Results

In both probands, a compound heterozygous mutations in *GLI3* gene were identified: NM_000168.6: c.[2179G>A];[4039C>A] (NP_000159.3: p.[Gly727Arg];[Gln1347Lys]), affecting exon 14 and 15 (Fig. 1d). According to American College of Medical Genetics (ACMG) criteria, variants were classified as uncertain significance because of very rare frequency in population databases (dbSNP rs121917710 and rs1441813326, GnomAD MAF 0.007131 and 0.00003187). We demonstrated high evolutionary conservation of both variants by positive scores in PhyloP (5,824/4,195) and phastCons (1/1), confirming slower evolution than expected under neutral drift. In order to check allelic configuration and confirm WES findings, we performed targeted sequencing on probands’ siblings. This examination indicated that these are *trans* variants, being located on different alleles. In daughters of proband II:3 we identified an occurrence of both mutations as a single variant (p.Gly727Arg in III:1 and p.Gln1347Lys in III:2). The daughter of second proband (III:3) showed the presence of p.Gly727Arg. All examined offspring had normal thyroid in US examination. Unfortunately, we could not check the mutation status in probands’ parents since they died years ago. Thus, an autosomal recessive transmission is inferred, which is a new and so far not reported for *GLI3* gene.

## Discussion

The *GLI3* is a gene located on 7p14.1 chromosome, encoding a protein of cytoplasmatic localization, known to play a key role in development which activates patched Drosophila homolog gene expression. This evolutionary conservative gene is expressed in multiple organs, mainly ovaries, placenta, endometrium, and presents moderate expression in the thyroid gland. The Gli3 protein is a transcription factor and negative regulator of Sonic hedgehog (Shh) signaling [8, 9], governing the symmetric lobulation of the thyroid. *Shh* gene knock-out mice present thyroid developmental failure resembling THA in humans [10], usually accompanied by other severe developmental anomalies, i.e., holoprosencephaly, trachea-esophageal fistula, and lung hypoplasia, while in humans such genetic variant is lethal [11]. Patients with Williams syndrome were also reported to have THA [1]. The microdeletion of 7q11.23 region in these patients harbors the *FZD9* gene that interacts with Shh and suggests contribution of this pathway. GLI3 is essential for human development. To date the role of GLI3 in embryogenesis was demonstrated for brain, lungs and limbs [9]. The *GLI3*-associated syndromes encompass polydactyly, central nervous system, and lungs pathologies, associating in Greig cephalopolysyndactyly and Pallister-Hall syndromes, presenting only autosomal dominant mode of inheritance [12]. In this report, none of the family members has clinical features of the mentioned GLI3-associated syndromes. Also, none of the probands’ offspring (obligatory heterozygotes) presented isolated THA.

This study represents the first report indicating a potential involvement of GLI3 in the thyroid development. The pathogenic effect on phenotype was documented for variant p.Gly727Arg (HGMD: CM990705) affecting an evolutionary conserved locus in the gene and segregating in a familial case of postaxial polydactyly being present in all affected family members [12]. Even though seven relatives were manifesting polydactyly and condition showed autosomal dominant inheritance, the variant was reported in population databases as very rare and therefore was classified as variant of unknown significance (VUS).

In our study, we deny its major causative impact also in regard of THA as a single separate factor (heterozygous *GLI3* allele defect in healthy offspring of probands). The second alteration p.Gln1347Lys is located 233 amino acid residues apart from the end of *GLI3* canonical transcript, before two independent transactivation domains TA1 (aa 1376–1580) and TA2 (aa 1044–1322) [13]. The change is predicted to be pathogenic in all used algorithms, and VUS according to ACMG (one control individual was reported for this position, but presenting different nucleotide substitution). More distally located mutations (AA1359–1486) were reported to be causative for at least seven cases of severe autosomal dominant Greig cephalopolysyndactyly syndrome [14]. This change was detected also in probands’ daughter who had normal thyroid on US. The case of compound heterozygosity has never been evidenced for *GLI3* and apparently milder THA phenotype detected in our study is surprising. However, we could not perform thyroid US in two out of five probands’ offspring. This constitutes a limitation of the study, same as that unaffected siblings of the probands refused to undergo genetic testing.

Mutations in exon 7 of GLI3 have been demonstrated also to cause other congenital malformation characterized by hypoplastic or absent tibia–hemimelia. Multiple similarities between THA and hemimelia should be mentioned here: occurs unilaterally, often as an isolated condition (rarely in syndromic cases), while family history is usually negative. Deimling et al. reported an exon 7 deletion of GLI3 in two patients with bilateral hemimelia, which causes truncated GLI3 protein that is deprived of DNA-binding domain and unable to repress SHH activity to the limb bud [15]. Interestingly, an asymptomatic mother of the proband was a carrier of the deletion, thus, authors came to a conclusion that tibial hemimelia may be inherited in an autosomal dominant mode with incomplete penetrance.

As potential mechanism how mutation of *GLI3* affects the SHH signaling we postulate a disequilibrium between the repressor and activator forms of the gene, what leads to lack of negative regulation of Shh. Shh is mostly expressed in epithelia therefore regulatory disturbances would have an effect particularly in organs derived from that kind of tissue. As evidenced for pituitary development [16, 17], the Shh pathway significantly contributes to the processes of morphogenesis and cell specification but both processes are not coupled. The consequences of emanation of the signals throughout developing tissue include not only a presence of a certain molecules but also an amount of signals that is locally expressed. Although in case of the reduction (or even absence) of Gli3 regulatory function, several alternative signaling cascades can be triggered (other Gli family molecules, i.e., Gli2 as well as recently evidenced Glis3) in order to compensate lack of Gli3. That would explain disturbances in thyroid shaping while maintaining the proper function of the tissue.

Interestingly, another associated transcription factor contributing to the Shh pathway—*GLIS3* (GLI-Similar protein 3) has recently been demonstrated to influence thyroid development. GLIS3 zebrafish knock-out shows a reduced number of thyroid follicles and an increase of the TSHβ subunit, resembling THA [18]. In humans, homozygous mutations in *GLIS3* lead to a multiple malformation syndrome encompassing CH. Rare heterozygous *GLIS3* missense variants were identified in 18 out of 177 patients with CH; half of them presented TDA (agenesis, hypoplasia, or ectopy) [19]. It was demonstrated that GLIS3 may bind to the GLI consensus sequence GACCACCCAC, what suggests a possible cross-talk between the GLI and GLIS3 signaling pathways [20].

Comparison of mice Gli3 and human GLI3 reveals that all domains demonstrate high similarity except the transactivation domain. Our mutations were identified in a proteasomal cleavage site of the protein (p.Gly727Arg) that showed 95% evolutionary conservation and the second one p.Gln1347Lys in a region bearing activatory function, presenting 76% similarity.

GLI3 haploinsufficiency has been noted in a wide range of human syndromes with a diverse severity, while phenotypic variability and incomplete penetration may be explained by coexistent mutations of SHH regulators. This may be one of the explanations for lack of extremities malformations, but the presence of an inherited THA in our family. Several mouse phenotypes associated with mutations of Gli3 affected the skeleton, eye, tail, extra toes, brachyphalangy, anterior digit deformity, and polydactyly. According to Radhakrishna et al. these mutants display phenotypic variability of expression, and there is no clear effect of the site of mutation on the phenotype. The authors proposed that all phenotypes associated with GLI3 mutations may be called “GLI3 morphopathies”. Hence there is no evident phenotype-genotype correlation of GLI3 mutations [12]. According to the results of our study, GLI3 morphopathies may include THA.

## Conclusions

We demonstrated for the first time a unique association of THA phenotype and the presence of compound heterozygous mutations p.[Gly727Arg];[Gln1347Lys] of *GLI3* gene in two siblings.
